# Candidate Gene Analysis for Nitrogen Absorption and Utilization in Japonica Rice at the Seedling Stage Based on a Genome-Wide Association Study

**DOI:** 10.3389/fpls.2021.670861

**Published:** 2021-06-04

**Authors:** Wei Xin, Jingguo Wang, Jia Li, Hongwei Zhao, Hualong Liu, Hongliang Zheng, Luomiao Yang, Chao Wang, Fan Yang, Jiahui Chen, Detang Zou

**Affiliations:** Key Laboratory of Germplasm Enhancement, Physiology and Ecology of Food Crops in Cold Region, Ministry of Education, Northeast Agricultural University, Harbin, China

**Keywords:** nitrogen use efficiency, japonica rice, *OsNAC68*, RNA-seq, genome-wide association study

## Abstract

Over-application of nitrogen (N) fertilizer in fields has had a negative impact on both environment and human health. Domesticated rice varieties with high N use efficiency (NUE) reduce fertilizer requirements, enabling sustainable agriculture. Genome-wide association study (GWAS) analysis of N absorption and utilization traits under low and high N conditions was performed to obtain 12 quantitative trait loci (QTLs) based on genotypic data including 151,202 single-nucleotide polymorphisms (SNPs) developed by re-sequencing 267 japonica rice varieties. Eighteen candidate genes were obtained by integrating GWAS and transcriptome analyses; among them, the functions of *OsNRT2.4*, *OsAMT1.2*, and *OsAlaAT* genes in N transport and assimilation have been identified, and OsJAZ12 and OsJAZ13 also play important roles in rice adaptation to abiotic stresses. A NUE-related candidate gene, *OsNAC68*, was identified by quantitative real-time PCR (qRT-PCR) analyses. *OsNAC68* encodes a NAC transcription factor and has been shown to be a positive regulator of the drought stress response in rice. Overexpression of *OsNAC68* significantly increased rice NUE and grain yield under deficient N conditions, but the difference was not significant under sufficient N conditions. NUE and grain yield significantly decreased under both N supply conditions in the *osbnac68* mutant. This study provides crucial insights into the genetic basis of N absorption and utilization in rice, and a NUE-related gene, *OsNAC68*, was cloned to provide important resources for rice breeding with high NUE and grain yield.

## Introduction

Rice is the most important food crop in the world, and sustainable and healthy crop development is important for global food security. Under the severe reality of increasing populations and decreasing land resources, the rice yield per unit area has been steadily enhanced by improving rice varieties, supporting advanced cultivation techniques, and increasing production investment. Nitrogen (N) is an important limiting element in rice production and plays an important role in the growth and yield of rice. However, behind the substantial increase in rice yield, excessive and unreasonable N fertilizer input has also incurred a series of problems, such as the decline in nitrogen use efficiency (NUE), the increase in production costs, and air and water pollution, which seriously affect sustainable rice production. How to reduce N fertilizer input in agricultural production and continuously increase crop yield has become a major issue in sustainable agricultural development.

In recent years, many NUE-related genes in rice have been discovered by quantitative trait locus (QTL) mapping and genome-wide association studies (GWASs) (Xu et al., 2012; Li et al., 2017). Through cloning and functional identification of these genes, the in-depth study of their mode of action and regulatory mechanisms not only has laid a good foundation for improving rice NUE-related research but also has important theoretical significance and practical application value (Gao et al., 2019, 2020; Tang et al., 2019; Guo et al., 2020; Yu et al., 2020).

Glutamate synthase is a key enzyme in rice N metabolism. Overexpression of its coding gene (*OsNADH-GOGAT1*) can increase spikelet grain weight and improve NUE of rice (Yamaya et al., 2002). Overexpression of *OsPTR9* can increase N uptake efficiency and yield in rice under normal growth conditions (Fang et al., 2013). Sun et al. (2014) showed that *DEP1*, a key gene for the increase of super rice yield in China, also plays a vital role in improving NUE of rice. Rice with *dep1-1* allelic variations is N-insensitive in vegetative growth and has increased N uptake and assimilation. The natural variation of a base in the nitrate transporter, NRT1.1b, can increase NUE in indica rice, and the introduction of indica *OsNRT1.1b* into japonica varieties can improve its NUE and yield (Hu et al., 2015). The functional study of the *OsNRT1.1b* homologous gene, *OsNRT1.1a*, showed that the yield and NUE of *OsNRT1.1a*-overexpression lines were significantly improved under low and high N conditions (Wang et al., 2018a). *GRF4* is the key gene of the gibberellin signal transduction pathway, which can promote N absorption and utilization and promote plant photosynthesis, thereby promoting plant growth and development. The high-level accumulation of the GRF4 protein can synergistically improve crop photosynthesis and NUE, so that rice can achieve higher yields with appropriate reduction in N fertilizer application (Li et al., 2018). NGR5 is a target of the gibberellin receptor, GID1, in promoting proteasome damage. Increasing NGR5 activity can improve NUE and yield in rice (Wu et al., 2020).

In summary, it is of great theoretical significance and practical application value to explore NUE-related genes in rice, study their function and expression characteristics, and analyze their molecular mechanisms. In this study, a rice NUE-related gene, *OsNAC68*, was identified by integrating GWAS and transcriptome analyses. *OsNAC68-*overexpression, transgenic rice (133-7, 133-11) and CRISPR/Cas9 gene editing knock-down expression mutant, *osnac68* (131-13, 131-36), were used as experimental materials to carry out the current study. The function of *OsNAC68* in rice NUE and yield formation was analyzed.

## Materials and Methods

### Plant Materials and Genotyping

The natural population for the GWAS comprised 267 japonica rice varieties, which were provided by the Institute of Crop Science, Chinese Academy of Agricultural Sciences. These varieties come from 45 countries and regions including China, Japan, the United States, etc.; all 267 japonica rice varieties included temperate japonica and tropical japonica rice (Supplementary Table 1). The high-density single-nucleotide polymorphism (SNP) loci were obtained from the “3K RG 4.8mio”-filtered SNP dataset (Wang et al., 2018b). A total of 151,202 SNPs were obtained for GWAS analysis by removing rare alleles [minor allele frequency (MAF < 5%)] and SNP markers with more than 20% missing markers (Supplementary Figure 1). The genetic structure of the population was calculated using STRUCTURE 2.3.4 software; the K (population) was preset to 1–10, and the length of the burn-in period at the beginning of Markov chain Monte Carlo (MCMC) was set to 10,000 times and then to “no counting.” The MCMC after iteration was set to 10,000, and then the appropriate population (K) value was selected according to the principle of maximum likelihood (Pritchard et al., 2000).

### Evaluation of N Absorption and Utilization Traits

Rice seeds were air-dried naturally and kept at 55°C for 5 days to break dormancy. They were then surface-sterilized with 1% sodium hypochlorite solution for 10 min, rinsed with sterile deionized water, and soaked in distilled water at 30°C in dark conditions for 2 days. The seeds were grown in a N-sufficient nutrient solution in a growth chamber (28/25°C; 10 h light/14 h dark). At the three-leaf heart stage, seedlings with the same growth were selected and supplied with 0.96 (Low-N) or 2.88 mMol L^–1^ ppm (High-N) of N using NH_4_NO_3_ as the source and grown under natural conditions for 30 days during the growing season. Each group included 30 seedlings. The hydroponic nutrient solution was formulated according to a previous research (Xin et al., 2019a). After 30 days of low and high N treatments, the above-ground part was harvested to determine N accumulation under low N conditions (LNA, mg plant^–1^), N accumulation under high N conditions (HNA, mg plant^–1^), NUE under low N conditions (LNUE, g g^–1^), and NUE under high N conditions (HNUE, g g^–1^). The samples were dried at 80°C to a constant weight, and shoot biomass was measured with a balance. Samples were then powdered with a micro-pulverizer (FZ102, China), and an element analyzer (Elementar Vario MACRO cube, Germany) was used to determine N contents. The heritability calculation of phenotypic data was conducted using lme4 (R package). NUE (g g^–1^) was calculated as:

(1) $$\mathrm{NUE}\left(gg^{-1}\right)=\mathrm{dry}\mathrm{matter}\mathrm{accumulation}\left(g\right)/N\,\mathrm{accumulation}\,\left(g\right).$$

### GWAS Analysis

GWAS was conducted *via* the mixed linear model (MLM) method using Tassel 5.0 software (Bradbury et al., 2007). The population structure (Q) and kinship calculated among individuals were used to adjust the population structure. The threshold was set at *P* < 3.31 × 10^–6^ (0.05/151,202) by the Bonferroni correction method (Li et al., 2019). To obtain the loci with the lowest *P*-value, redundant SNPs were filtered in a least-distance (LD) interval, and the SNP with the lowest *P*-value was considered the lead SNP. The Manhattan plot and quantile–quantile (Q–Q) plot were produced using the “CMplot” package in R.

### Candidate Gene Prediction

The region of lead SNPs ± 100 kb was defined as the candidate region for NUE. The gene annotation and RNA sequencing data that were collected (Supplementary Table 2) under low and control N conditions in previous studies (Xin et al., 2019a,b) were combined to predict candidate genes.

### RNA Extraction and Quantitative Real-Time PCR Analysis

For analysis of candidate gene expression in leaves, six N-inefficient and N-efficient varieties were selected according to the N absorption and utilization traits in 267 japonica rice varieties under low and high N conditions. The procedure and management of the experiment were the same as those in the above-mentioned experiment. Total RNA was extracted from rice leaves using the TransZol Up RNA Kit (TransGen Biotech, Beijing, China). Complementary DNA was synthesized from total RNA using the HiFiScript cDNA Synthesis Kit (CWBio, Beijing, China). Quantitative real-time PCR (qRT-PCR) analysis was performed according to a previous study (Xin et al., 2019a). All primer information is shown in Supplementary Table 3.

### Vector Construction and Plant Transformation

The CRISPR/Cas9 gene editing vector construction was conducted as described by Li et al. (2017). Two target sequences (including PAM) (TACTTGCCGGTGAGGTCGTCGGG, ACCGTGCGGTCCAAGACACCGG) were selected within the target genes, and their targeting specificity was confirmed using a BLAST search against the rice genome (Hsu et al., 2013). Rice transformation was performed as described previously (Nishimura et al., 2006). Genomic DNA was extracted from these transformants, and primer pairs flanking the designed target site were used for PCR amplification. The PCR products (300–500 bp) were sequenced directly and identified using the Degenerate Sequence Decoding method (Ma et al., 2015). Knockout lines were confirmed by PCR sequencing with primers 5′-CCGCCGACTTCGGCTCCC-3′ and 5′-GGGAGGTGGGGCGGCCATG-3′ (Supplementary Figure 2). The cDNA was cloned into the pBWA(V) HS vector between the 35S promoter and terminator, generating a 35S::NAC68 construct. *OsNAC68* amplification occurred with primers 5′-CAGTGGTCTCACAACATGTCCCCCTCCCGCCCC-GACG-3′ and 5′-CGATGGTCTCACAAGAACCTGATGAATTTGCCA-3′ (Supplementary Figure 3).

### Determination of Yield and NUE of Genetically Modified Materials

The control rice, “Shennong 9816” (wild-type, WT), *OsNAC68*-overexpression (133-7, 133-11) lines, and *osnca68*-mutant (131-13, 131-36) rice were planted in pots (inner diameter 30 cm). The experiment was conducted at Northeast Agricultural University, China in 2020. The tests included two N application rate treatments, 60 kg (pure nitrogen) ha^–1^ (deficient nitrogen, DN) and 180 kg (pure nitrogen) ha^–1^ (sufficient nitrogen, SN), using urea as the source (46% N content). N fertilizer was applied with basal, tillering, and panicle fertilizer at a ratio of 6:3:1 at the relevant growth stage. Phosphate fertilizer (P_2_O_5_) was applied once as a basal fertilizer at a rate of 90 kg ha^–1^. Potash (K_2_O) fertilizer was applied as a basal and panicle fertilizer at a ratio of 5:5, at a rate of 90 kg ha^–1^. With the exception of the different N fertilizer application rates, the other cultivation requirements were identical for all pots. In the mature stage, the yield per plant, the number of effective ears per plant, the number of grains per ear, the seed setting rate, and the thousand-grain weight were calculated. The rice plants were divided into shoots and panicles at the maturity stage, and they were killed at 105°C for 30 min, dried at 80°C to a constant weight, and weighed to determine the accumulation and distribution of dry matter. The dried tissues were ground into powder and passed through a 100-mesh sieve. The N concentration of each tissue was measured with an elemental analyzer to calculate the total N accumulation (TNA). N utilization efficiency for biomass production (NUpE, g g^–1^) was calculated as:

(2) NUpE(gg)-1=Biomassproductionatmaturity(g)/Total⁢N⁢accumulation⁢at⁢maturity⁢(g).

N utilization efficiency for grain production (grain yield) (NUgE, g g^−1^) was calculated as:

(3) $$\mathrm{NUgE}\,\left(g\,g^{-1}\right)=\mathrm{Grain}\,\mathrm{yield}\,\left(g\right)/\mathrm{Total}\,N\mathrm{accumulation}\,\mathrm{at}\,\mathrm{maturity}\,\left(g\right).$$

## Results

### Phenotypic Variation in the Natural Population

**FIGURE 1:**
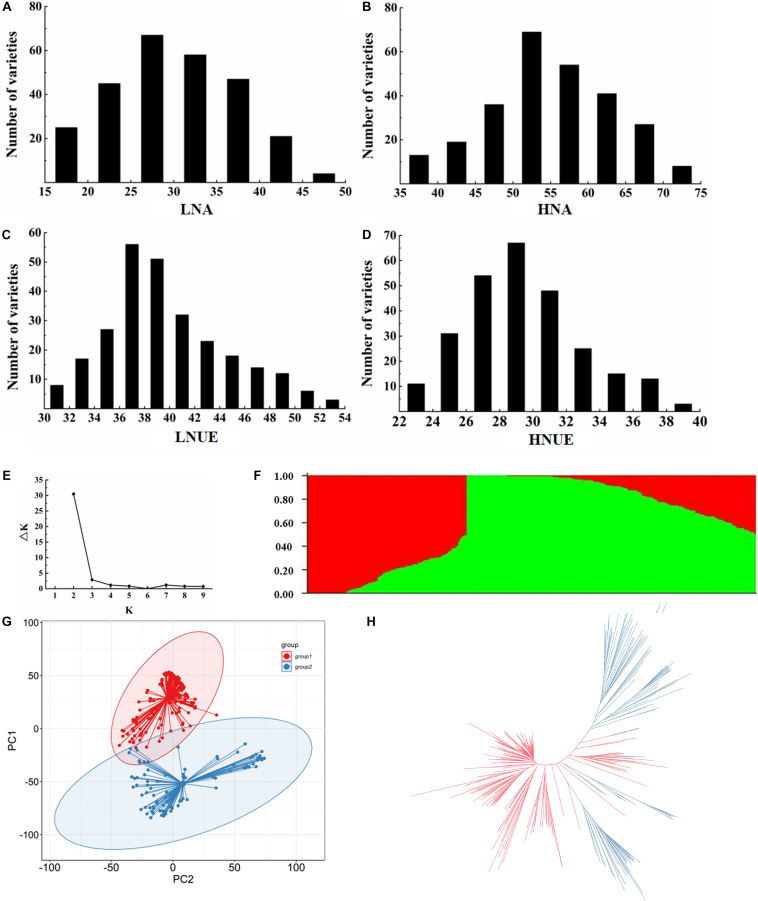
Phenotypic variation and population structure in 267 japonica rice varieties. **(A–D)** represent the LNA, HNA, LNUE, and HNUE of natural populations, respectively. **(E)** The variation curve of ΔK with K value. **(F)** Subgroups (*K* = 2) inferred using ADMIXTURE software. **(G)** Principal component analysis of 267 japonica rice varieties. Colors of red and blue represent G1 and G2 in **(F)**, respectively. **(H)** Neighbor-joining tree of 267 japonica rice varieties. Colors of red and blue represent G1 and G2 in **(F)**, respectively.

To assess the phenotypic variation in NUE in 267 japonica rice varieties at the seedling stage, four NUE-related traits were evaluated: LNA, HNA, LNUE, and HNUE. The means, standard deviations, and ranges of LNA, HNA, LNUE, and HNUE at the seedling stage of natural populations are presented in Supplementary Table 4. The heritability of the NUE-related traits was higher (68.3–77.2%), indicating that population NUE-related traits are largely affected by genetic factors. The mean LNA was lower than HNA, and the mean LNUE was higher than HNUE, indicating that under low N conditions, the N acquisition of rice from the growth environment was limited, and the N utilization ability of rice to adapt to low N stress was strengthened. Statistical analysis (Figure 1) revealed continuous variation in each trait, which was consistent with the genetic characteristics of quantitative traits controlled by multiple genes. In addition, based on these traits, we identified six N-inefficient varieties and six N-efficient varieties in the natural population (Supplementary Table 5).

### SNP Validation and Population Structure Analysis

The high-density SNP loci were obtained from the “3K RG 4.8mio”-filtered SNP dataset (Wang et al., 2018b). A total of 151,202 SNPs were obtained for GWAS, distributed on 12 chromosomes, with an average of 12,600.17 SNPs per chromosome. The genetic structure of 267 japonica germplasm resources was stratified (Figure 1E). When *K* = 2, the population was divided into two subgroups, designated G1 (temperate japonica) and G2 (tropical japonica). Principal component analysis and phylogenetic analysis are shown in Figures 1G,H.

### GWAS for N Absorption- and Utilization-Related Traits in a Natural Population

**FIGURE 2:**
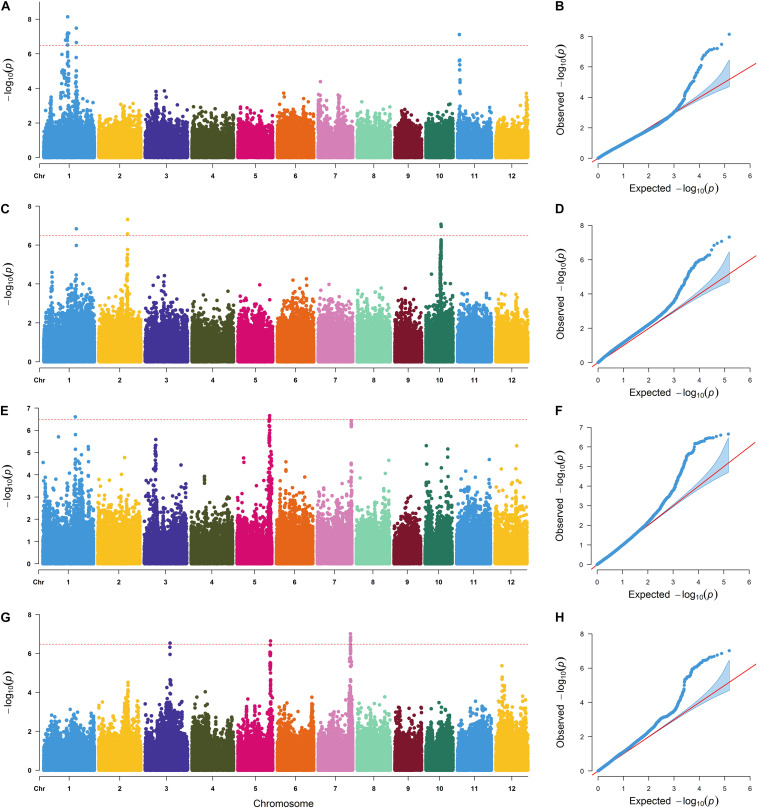
Manhattan plots and quantile–quantile (Q–Q) plots of genome-wide association studies for the LNA, HNA, LNUE, and HNUE. **(A)** Manhattan plot for the LNA. **(B)** Q–Q plot for the LNA. **(C)** Manhattan plot for the HNA. **(D)** Q–Q plot for the HNA. **(E)** Manhattan plot for the LNUE. **(F)** Q–Q plot for the LNUE. **(G)** Manhattan plot for the HNUE. **(H)** Q–Q plot for the HNUE.

GWAS was performed *via* the MLM method, with principal component analysis and kinship matrix (PCA + K) as covariates to correction of the population structure, in Tassel 5.0 software. The Manhattan and Q–Q plots for the GWAS results are shown in Figure 2. Taking −log_10_ (P) ≥ 6.25 as the threshold, 12 lead SNPs significantly associated with LNA, HNA, LNUE, and HNUE are listed in Table 2. These SNPs were located on chromosomes 1, 2, 3, 5, 7, 10, and 11 with −log_10_ (P) values ranging from 6.284 to 8.052. Three significant lead SNPs for the LNA were distributed on chromosomes 1 and 11 and were named *qLNA1-1*, *qLNA1-2*, and *qLNA11*, respectively. Three significant lead SNPs for the HNA were distributed on chromosomes 1, 2, and 10 and named *qHNA1*, *qHNA2*, and *qHNA10*, respectively. Three significant lead SNPs for the LNUE were distributed on chromosomes 1, 5, and 7 and named *qLNUE1*, *qLNUE5*, and *qLNUE7*, respectively. Three significant lead SNPs for the HNUE were distributed on chromosomes 3, 5, and 7 and named *qHNUE3*, *qHNUE5*, and *qHNUE7*, respectively (Figure 2 and Table 1). These lead SNPs are critical for N absorption and utilization at the seedling stage of rice, and there may be candidate genes for N absorption and utilization nearby.

**TABLE 1:** Lead SNPs for LNA, HNA, LNUE, and HNUE identified by GWAS.

**Traits**	**QTLs**	**Lead SNPs**	**Chr**	**Position**	**−Log_10_ (P)**
LNA	*qLNA1-1*	rs1_20390767	1	20390767	8.052
	*qLNA1-2*	rs1_27777202	1	27777202	8.000
	*qLNA11*	rs1_1072051	11	1700590	7.135
HNA	*qHNA1*	rs1_27777202	1	27777202	7.312
	*qHNA2*	rs1_29282639	2	24692377	7.280
	*qHNA10*	rs1_18147798	10	13016418	7.264
LNUE	*qLNUE1*	rs1_27750585	1	27750585	7.704
	*qLNUE5*	rs1_23142555	5	27948915	6.518
	*qLNUE7*	rs1_24796548	7	29143588	6.284
HNUE	*qHNUE3*	rs1_12340227	3	21308046	6.638
	*qHNUE5*	rs1_23089121	5	27856627	6.336
	*qHNUE7*	rs1_24144900	7	27684185	7.241

### Candidate Gene Analysis Using GWAS and RNA-seq

**FIGURE 3:**
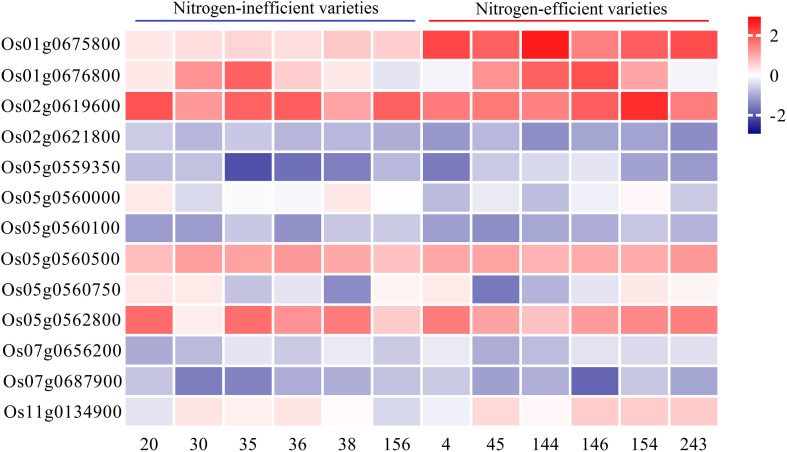
Relative expression of candidate genes. qRT-PCR verification of the transcript levels of candidate genes under low and high N conditions.

A total of 271 genes were retrieved from the above 11 SNPs ± 100 kb region (Supplementary Table 6), and 18 candidate genes (Table 2) were obtained by combining the previously reported RNA sequencing data of rice leaves and roots under low N conditions (Supplementary Table 2). The functions of the *OsNRT2.4*, *OsAMT1-2*, and *OsAlaAT* genes in N transport and assimilation have been identified (Kikuchi et al., 1999; Sonoda et al., 2003; Feng et al., 2011). *OsJAZ12* and *OsJAZ13* also play important roles in rice adaptation to abiotic stresses (Ye et al., 2009). Through further integrated analysis, we selected 13 candidate genes to compare expression levels between N-inefficient and N-efficient varieties using qRT-PCR analysis under low and high N conditions (Figure 3). The qRT-PCR results showed that the expression levels of *Os01g0675800* in different rice varieties were significantly different, and that *Os01g0675800* showed higher expression levels in N-efficient varieties than in N-inefficient varieties under both N conditions (Supplementary Table 7). *Os01g0675800* was near *qLNA1-2*, *qLNA1-2*, and *qLNUE1*. These results suggest that *Os01g0675800* is likely to be a candidate gene for *qLNA1-2*, *qHNA1*, and *qLNUE1*.

**TABLE 2:** Candidate genes differentially expressed under LN conditions.

**Candidate genes**	**Root**	**Leaf**	**QTLs**	**Known genes**
Os01g0547600	Up	Up	*qLNA1-1*	*OsNRT2.4* (Kikuchi et al., 1999)
Os01g0675800	Up	Up	*qLNA1-2*, *qHNA1*, *qLNUE1*	
Os01g0676800	Up	Up	*qLNA1-2*, *qHNA1*, *qLNUE1*	
Os02g0619600	Up	Up	*qHNA2*	
Os02g0620600	Up	Down	*qHNA2*	*OsAMT1.2* (Sonoda et al., 2003)
Os02g0621800	Down		*qHNA2*	
Os05g0559350	Down	Down	*qHNUE5*	
Os05g0560000	Down		*qLNUE5*, *qHNUE5*	
Os05g0560100	Down	Down	*qLNUE5*, *qHNUE5*	
Os05g0560500	Up		*qLNUE5*, *qHNUE5*	
Os05g0560750		Down	*qLNUE5*, *qHNUE5*	
Os05g0562800	Up		*qLNUE5*	
Os07g0656200	Down		*qHNUE7*	
Os07g0687900		Down	*qLNUE7*	
Os10g0390500		Down	*qHNA10*	*OsAlaAT* (Feng et al., 2011)
Os10g0391400	Up	Up	*qHNA10*	*OsJA13* (Ye et al., 2009)
Os10g0392400	Up	Up	*qHNA10*	*OsJA12* (Ye et al., 2009)
Os11g0134900		Down	*qLNA11*	

### Effects of *OsNAC68* on Grain Yield Under Different N Levels

**FIGURE 4:**
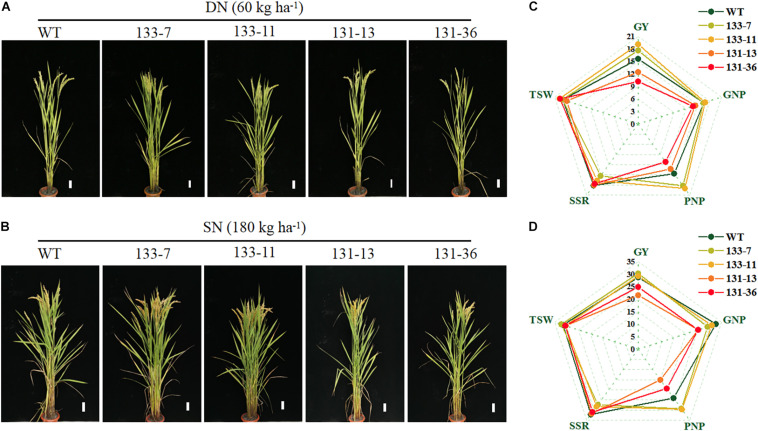
Effects of *OsNAC68* on phenotypes and grain yield of rice under different nitrogen fertilizer levels. **(A,B)** Phenotypes of the whole rice plants WT and transgenic lines under DN and SN conditions at the ripening stage. Scale bar, 5 cm. **(C,D)** Grain yield of the WT and transgenic lines under DN and SN conditions. GY, grain yield; GNP, grain number per panicle; PNP, panicle number per plant; SSR, seed setting rate; TSW, 1,000 grain weight.

*Os01g0675800* encodes a NAC transcription factor (*OsNAC68*), which has been shown to be a positive regulator of drought response in rice (Shim et al., 2018). To confirm the function of *OsNAC68* on grain yield and NUE of rice, we generated *osnac68-*mutant (131-13, 131-36) lines using the CRISPR/Cas9 method and generated *OsNAC68*-overexpression (133-7, 133-11) lines in which the *OsNAC68* gene was driven by the 35S promoter. Compared with the WT, the yield per plant of *OsNAC68-*overexpression transgenic rice lines increased by 17.88 and 4.72% under DN and SN conditions, respectively, whereas the yield per plant of *osnac68* mutant decreased by 21.15 and 17.97% under DN and SN conditions, respectively (Figure 4 and Supplementary Table 8). The yield-related traits analysis showed that the effective panicle number per plant of *OsNAC68*-overexpression transgenic rice was significantly higher than that of the WT under the two N supply levels, but the seed setting rate was significantly decreased. The grain number per panicle and 1,000 grain weight were not significantly different compared with the WT. Under both N supply levels, the grain number per panicle and the effective panicle number per plant of the *osnac68* mutant were significantly lower than those of the WT, and the 1,000 grain weight and seed setting rate decreased, but the difference was not significant compared with the WT (Figure 4 and Supplementary Table 8).

### Effects of *OsNAC68* on N Uptake and Utilization in Rice Under Different N Levels

**FIGURE 5:**
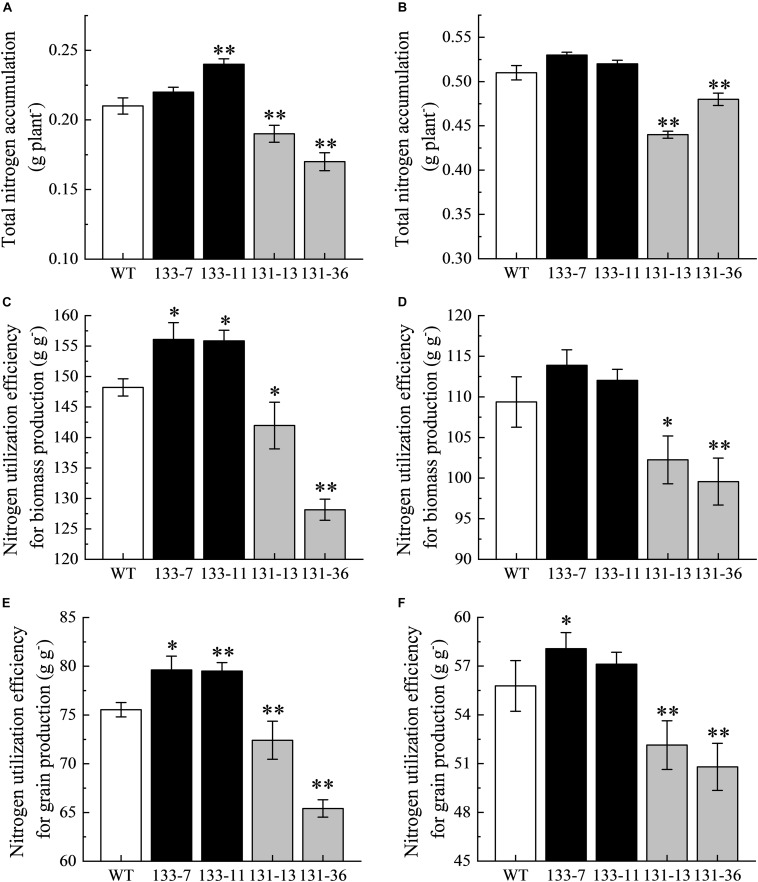
Evaluation of total nitrogen accumulation (TNA), nitrogen utilization efficiency for biomass production (NUpE), and nitrogen utilization efficiency for grain production (NUgE) in transgenic rice. **(A,B)** TNA under DN and SN conditions. **(C,D)** NUpE under DN and SN conditions. **(E,F)** NUgE under DN and SN conditions. Values are mean ± SD (*n* = 5), ^∗^*P* < 0.05, ^∗∗^*P* < 0.01 (*t*-test).

Compared with the WT, overexpression of *OsNAC68* significantly increased TNA (except 133-7), NUpE, and NUgE, whereas the *osnac68* mutant significantly decreased TNA, NUpE, and NUgE under DN conditions (Figure 5). Compared with the WT, overexpression of *OsNAC68* increased TNA, NUpE, and NUgE (except 133-7), but the difference was not significant, whereas the *osnac68* mutant significantly decreased TNA, NUpE, and NUgE under SN conditions.

## Discussion

N is present in the substances necessary for plant growth and development, such as proteins, amino acids, and phytohormones. These substances participate in physiological and metabolic regulation in plants and control various life activities and yield formation of plants. Too much or too little use of N fertilizer will cause economic losses. Improving the NUE of plants can reduce the amount of N fertilizer applied, which is essential for reducing agricultural costs, controlling environmental pollution, and ensuring food security. NUE is a measure of the difference between obtaining N from the environment and using it for growth and development (Garnett et al., 2009; Nunes-Nesi et al., 2010). An ideal, N-efficient variety needs to absorb more N from the environment and use the absorbed N to produce more biomass and grain yield.

This study shows that under low and high N conditions, different rice varieties have significant genotypic differences in N absorption and utilization efficiency, which is consistent with previous studies (Zhang et al., 2009; Ju et al., 2015). Previous studies have shown that N absorption and utilization are controlled by different genes (Broadbent et al., 1987; Datta and Broadbent, 1990). Under different levels of N supply, the genetic basis of N-efficiency of rice is related to N absorption and utilization (Senthilvel et al., 2008). Therefore, in this study, the N absorption and utilization indicators (LNA, LNUE, HNA, and HNUE) under low and high N supply were used as the NUE-related traits for GWAS analysis.

NUE is a complex quantitative trait, controlled by major genes and multiple, minor QTLs. In recent years, with the advances in molecular biology, many NUE-related genes have been identified, such as *OsDEP1*, *OsNRT1.1B*, *OsNAC42*, *OsNPF6.1*, *OsNLP4*, *OsGRF4*, *OsNGR5*, *OsDNR1*, and *OsARE1* (Sun et al., 2014; Hu et al., 2015; Li et al., 2018; Wang et al., 2018; Tang et al., 2019; Wu et al., 2020; Yu et al., 2020). These studies provide a new breeding strategy for the sustainable agricultural development of rice and other crops with “less input, more output, and environmental protection,” which is of great significance both in theory and in application. In this study, 12 QTLs were identified under low and high N conditions. Only two QTLs (SNP_27750585, SNP_27777202) were co-located in NA and NUE traits, indicating that N absorption and utilization have a specific genetic basis. The genes *qLNA1-2*, *qHNA1*, and *qLNUE1* are at the same or near these sites, as are *qLNUE5* and *qHNUE5*. The results of the QTL interval gene annotation showed that there were no known genes related to N absorption, utilization, and regulation in the interval. This suggested that unknown NUE-related genes are highly likely to exist within the QTL interval, and that these genes are necessary to maintain normal plant growth, so should be detectable under different N supply conditions.

Transcriptome analysis can be used to characterize the response of various plant species to environmental stresses (Wang et al., 2009). Many studies have shown that, compared with normal N conditions, many genes show differential expression, including N metabolism-related, stress resistance-related, and hormone-related genes. Under low N stress conditions, these differentially expressed genes (DEGs) play a crucial role in rice adaptation to low N stress (Yang S. et al., 2015; Yang W. et al., 2015; Xin et al., 2019a,b; Subudhi et al., 2020). In recent years, with the development of high-throughput sequencing technology and the improvement of the transcriptome database, the combination of transcriptome and traditional localization methods has been widely used in candidate gene prediction. Yu et al. (2020) identified a N-efficient gene, *OsNLP4*, by integrating NUE-related traits, GWAS analysis, and transcriptome data under N-sufficient and N-starved conditions. Yao et al. (2020) used integrated GWAS and transcriptome analysis methods to obtain a series of candidate genes (including *ZmSnRk2*, *ZmPYL*, *ZmNPR1*) for resistance to *Fusarium* ear rot in maize. Our previous research also identified many saline-alkaline-tolerant candidate genes (*OsIRO3*, *OsSAP16*) by integrating GWAS/BSA and transcriptome data analysis (Li et al., 2019; Lei et al., 2020). This study identified 18 potential NUE-related candidate genes by combining the previously reported RNA sequencing data of rice leaves and roots under low N conditions. The functions of *OsNRT2.4*, *OsAMT1-2*, and *OsAlaAT* genes in N transport and assimilation have been identified (Kikuchi et al., 1999; Sonoda et al., 2003; Feng et al., 2011). *OsJAZ12* and *OsJAZ13* also play important roles in rice adaptation to abiotic stresses (Ye et al., 2009). From further qRT-PCR analysis of varieties with different N use efficiencies, *Os01g0675800* was identified as a likely candidate gene for N absorption and utilization.

The gene, *Os01g0675800*, is a transcription factor of the NAC family. Shim et al. (2018) found that overexpression of *OsNAC68* significantly increased drought resistance in rice. The NAC transcription factor family is an important transcription regulatory factor, which is ubiquitous in plants. In the life course of rice, the NAC family is involved in cell growth, tissue development, organ aging, and other processes and plays an important role in the response to external environmental change (Jeong et al., 2010, 2013; Redillas et al., 2012; Lee et al., 2017; Chung et al., 2018). In addition, nitrate signaling regulates root growth through *AtNAC4* (Alvarez et al., 2020). In this study, the GY, NA, NUpE, and NUgE of the *OsNAC68*-overexpression lines were higher than those of the WT, especially under DN conditions. The GY, NA, NUpE, and NUgE of the *osnac68-*mutant lines were significantly lower than those of the WT under both N conditions. These results indicate that *OsNAC68* can mediate the process of N absorption and utilization in rice, thereby affecting rice yield formation. Previous studies found that *OsNAC42* can regulate *OsNPF6.1* and increase the N acquisition capacity of rice, thereby increasing rice yield (Tang et al., 2019). Ueda et al. (2020) found that *OsONAC1* and *OsNAC2* can regulate N metabolism-related modules under low N stress. Two transcription factors, *AtHAP2C* and *AtNAC096*, and an integrase-type DNA (*At4g39780*) were found to regulate 53% of N responsive genes throughout the root system as downstream of *AtNLP7* (Alvarez et al., 2020). Investigating whether *OsNAC68* interacts with the N absorption- and utilization-related genes cascade will help to reveal the molecular mechanism of N absorption and utilization in rice. It is important for the breeding of rice with a high NUE and yield.

## Conclusion

In the present study, natural populations were collected to evaluate the N absorption and utilization at the seedling stage. GWAS analysis of N absorption and utilization traits under low and high N conditions was performed to obtain 12 QTLs based on genotypic data including 151,202 SNPs developed by re-sequencing 267 japonica rice varieties. A NUE-related candidate gene, *OsNAC68*, was identified by GWAS, RNA-seq, and qRT-PCR analyses. The functions of *OsNAC68* in rice N absorption and utilization and yield were further verified through overexpression and gene editing technology. However, the regulation mechanism of *OsNAC68*-mediated N absorption and utilization in rice still needs further study. This study provides resources for breeding aimed at improving rice N absorption and utilization.

## Data Availability Statement

The original contributions presented in the study are included in the article/Supplementary Material, further inquiries can be directed to the corresponding author/s.

## Author Contributions

DZ and WX designed the study and provided experimental materials. WX analyzed the results, prepared the figures and tables, and wrote the manuscript. All authors discussed the results and commented on the manuscript, read and approved the final manuscript.

## Conflict of Interest

The authors declare that the research was conducted in the absence of any commercial or financial relationships that could be construed as a potential conflict of interest.

## Supplementary Material

Supplementary Figure 1 SNP density of 267 japonica rice population.

Supplementary Figure 2 Knockout of *OsNAC68* using CRISPR/Cas9 system. Sequence of wild-type transgenic line (WT) and the two mutational lines of OsNAC68 in the target region. Red letters indicated 1-bp insertions (131-13) and 4-bp deletion (131-36), Blue letters indicate the PAM region.

Supplementary Figure 3 The overexpression of *NAC68*. **(A)** Overexpression construct for rice transformation. **(B)** The expression levels of 133-7, 133-11, and wild-type (WT).

Supplementary Table 1 Geographical distribution of 267 japonica rice varieties.

Supplementary Table 2 Differentially expressed genes (DEGs) in leaves and roots under deficient nitrogen conditions compared with sufficient nitrogen conditions.

Supplementary Table 3 Primers for qRT-PCR in this study.

Supplementary Table 4 Phenotypic variation in 267 japonica rice varieties under alkalinity condition. SD, standard deviation, CV, coefficient of variation.

Supplementary Table 5 LNA, HNA, LNUE, and HNUE of nitrogen-inefficient and nitrogen-efficient varieties.

Supplementary Table 6 Annotated genes in the QTLs region.

Supplementary Table 7 qRT-PCR verification of the transcript levels of candidate genes under low and high N conditions.

Supplementary Table 8 Yield-related characteristics of WT and transgenic rice under DN and SN conditions. Values are means ± SD (*n* = 5), ^∗^*P* < 0.05, ^∗∗^*P* < 0.01 (*t*-test).
