# Supplementary figures and images for: Candidate Gene Analysis for Nitrogen Absorption and Utilization in Japonica Rice at the Seedling Stage Based on a Genome-Wide Association Study
Source: Front Plant Sci. 2021 Jun 4;12:670861. doi: 10.3389/fpls.2021.670861 (PMC8212024; doi:10.3389/fpls.2021.670861)

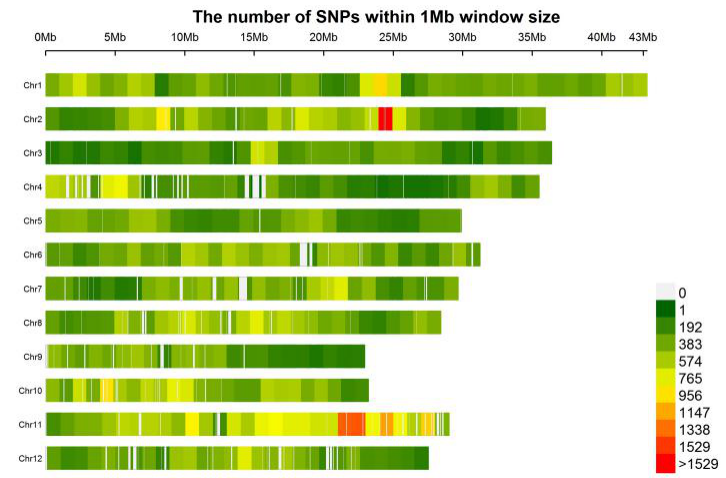

Supplement: Supplementary Figure 1 — SNP density of 267 japonica rice population. [file Image_1.TIF]

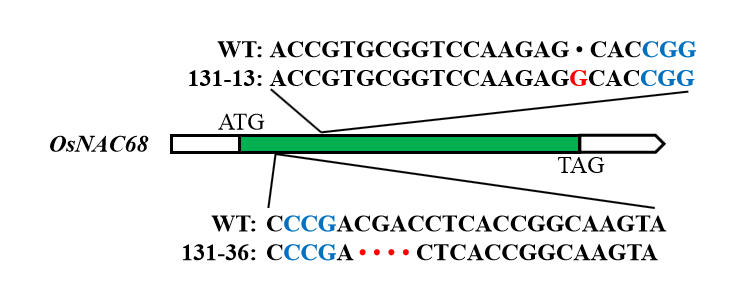

Supplement: Supplementary Figure 2 — Knockout of OsNAC68 using CRISPR/Cas9 system. Sequence of wild-type transgenic line (WT) and the two mutational lines of OsNAC68 in the target region. Red letters indicated 1-bp insertions (131-13) and 4-bp deletion (131-36), Blue letters indicate the PAM region. [file Image_2.TIF]

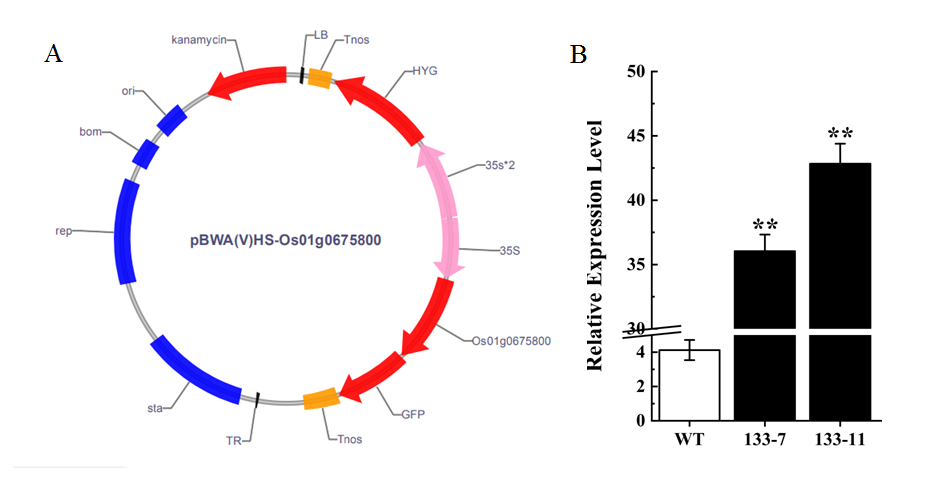

Supplement: Supplementary Figure 3 — The overexpression of NAC68. (A) Overexpression construct for rice transformation. (B) The expression levels of 133-7, 133-11, and wild-type (WT). [file Image_3.TIF]
